# Efficacy of 2% Paradichlorobenzene, 10% Citric Acid, 17% Ethylenediaminetetraacetic Acid, and 0.2% Chitosan at Removing Calcium Hydroxide From the Root Canals

**DOI:** 10.7759/cureus.49607

**Published:** 2023-11-28

**Authors:** Neha Mehra, Ankeeta Singh, Mamta Kaushik, Soujanya Goud, Sai Madhuri Battula

**Affiliations:** 1 Conservative Dentistry and Endodontics, Army College of Dental Sciences, Secunderabad, IND; 2 Conservative Dentistry and Endodontics, Dental Folks, New Delhi, IND; 3 Conservative Dentistry and Endodontics, Army College of Dental Sciences, Hyderabad, IND

**Keywords:** paradichlorobenzene, itk- snap, cone beam computed tomography, ceruminolytic agent, calcium hydroxide, calcium chelators

## Abstract

Introduction: Chronic infections often require the use of an intracanal medicament. Calcium hydroxide is the most commonly used intracanal medicament. However, retrieval of calcium hydroxide (CH) medicaments is a challenge.

Aim: This study evaluated the efficacy of 2% paradichlorobenzene, 10% citric acid, 17% ethylenediaminetetraacetic acid (EDTA), and 0.2% chitosan in removing calcium hydroxide from the root canals.

Methods and Materials: Forty single-rooted mandibular premolars extracted for orthodontic reasons were selected for the study. After access opening, cleaning and shaping were performed following a standardized irrigation protocol, and the samples were pre-weighed for baseline weight. CH was placed in canals until the apex, and the specimen was incubated at 37 °C and 100% relative humidity for seven days. These samples were weighed again for quantitative analysis and subjected to cone beam computed tomography (CBCT) for volumetric analysis using ITK SNAP software (Penn Image Computing and Science Laboratory (PICSL), University of Pennsylvania, Philadelphia, PA). The samples were randomly divided into four groups based on the irrigant used for the removal of medicament. Group 1: 2% paradichlorobenzene; group 2: 10% citric acid; group 3: 17% EDTA; and group 4: 0.2% chitosan, all in combination with sonic agitation. After the removal of CH, weight and volumetric analyses were repeated, and the percentage difference was calculated.

Statistical analysis: Statistical analysis was done using the one-way ANOVA test for both weight and volumetric assessment, and the inter-group comparison was made using the post hoc Tukey test.

Results: The maximum retrieval was observed with 2% paradichlorobenzene by both weight (96.75%) and volumetric (91.42%) assessment, with p=0.00 and p=0.01, respectively. This was followed by 0.2% chitosan, 10% citric acid, and the least, 17% EDTA.

Conclusion: Two percent paradichlorobenzene combined with sonic agitation was most efficient in removing CH, followed by 0.2% chitosan, 10% citric acid, and 17% EDTA chitosan.

## Introduction

Microorganisms and their by-products play a significant role in the development and progression of pulpal and periapical diseases [1,2]. The anatomic complexities of the root canal system, like the isthmus, lateral and accessory canals, and apical ramifications, contribute to the survival of bacteria in the dentinal tubules and root canals, resulting in persistent or secondary infections [2]. In such instances, the chemo-mechanical instrumentation must be supplemented with intracanal medicaments to facilitate complete disinfection of the root canal system [3].

Calcium hydroxide (CH) is the most frequently used intracanal medicament; it is biocompatible and has a profound antimicrobial effect. The alkaline pH causes the degradation of lipopolysaccharides, tissue dissolution, and inhibits osteoclastic activity [4]. CH is usually placed in the root canals for one to four weeks for disinfection [4,5].

The removal of medicament is essential prior to obturation, as the remnants may prevent the contact of sealer with dentinal tubules, interfere with the setting of certain root canal sealers, making them brittle and granular, and ultimately result in leakage after obturation [6]. CH also reduces the bond strengths of resin-based sealers to root canal dentine and may interfere with the sealing ability of a silicon-based sealer [7,8].

Various irrigants like saline, sodium hypochlorite (NaOCl), ethylenediaminetetraacetic acid (EDTA), maleic acid, and citric acid have been investigated for the removal of CH. EDTA in a 15-17% concentration is a root canal irrigant with chelating properties [9-13]. The property of chelation helps remove the smear layer from the root canal and aids in the removal of CH medicament by chelating calcium ions to EDTA [9,10].

Maleic acid (7%) efficiently removes CH from root canals due to its superior decalcifying effect [12]. Likewise, citric acid (20%) was also superior to EDTA for the removal of CH [9]. However, none of the mentioned irrigants facilitate the complete removal of CH from root canals [9,12,13].

Chitosan, a natural polysaccharide prepared by the deacetylation of chitin, is biocompatible, biodegradable, shows bioadhesion, and has minimal toxicity [14]. Its chelation activities are similar to those of EDTA and citric acid, with less detrimental effects [9]. Investigations to assess 0.2% chitosan have displayed significantly higher efficacy at removing CH from the root canals than 17% EDTA and 20% citric acid [9,13].

Cerumen (ear wax) softening agents, like 2% paradichlorobenzene, have a unique property to soften, disperse and dissolve earwax. Through successful disintegration, they reduce the wax plug’s natural integrity, and by standard migrating mechanisms, these smaller fragments are removed from the ear [15]. This property could also apply to soften or disintegrate CH in the root canals.

Hence, this study aimed to assess the efficacy of 2% paradichlorobenzene, 10% citric acid, 17% EDTA, and 0.2% chitosan for removing CH from the root canal. The null hypothesis was that there was no difference between these agents in removing calcium hydroxide from root canals.

## Materials and methods

Teeth selection

The manuscript of this laboratory study is written according to the Preferred Reporting Items for Laboratory Studies in Endodontology (PRILE) 2021 guidelines. The study protocol was approved by the Institutional Review Board with certificate number ACDS/IRB/03/JUN/2021, dated June 22, 2021. Using the G Power software (version 3.1.9.7) and data obtained from a previous study, a minimum sample size of 40 was established [13]. Forty freshly extracted human mandibular premolar teeth were collected from the Department of Oral and Maxillofacial Surgery and stored in distilled water at room temperature. The extracted teeth were evaluated with radiographs and under the Dental Operating Microscope (Prima; Labomed, Los Angeles, CA) for meeting the inclusion criteria. The inclusion criteria were single-rooted teeth and teeth with completely formed root apices. The exclusion criteria were teeth showing caries, previous endodontic treatment, root resorption, calcification, fracture, or cracks.

Sample preparation

Access cavities were prepared using Endo Access Bur (Dentsply Sirona, Switzerland), and apical patency was established using a size 10 K-file (Mani, Inc., Delhi, India).

The working length was determined with a 10 K file radiographically and confirmed under 6× magnification using a stereomicroscope (SMZ 800, Nikon, Tokyo, Japan), with the file tip just visible at the apical foramen. Chemo-mechanical preparation was performed with the crown-down technique using ProTaper Universal rotary files (Dentsply Sirona, Vaud, Switzerland) until size F3. A standardized irrigation protocol was followed using 2 ml of 3% NaOCl (Vishal Dentocare Pvt. Ltd., Ahmedabad, India) after each instrument and followed with 5 ml of 17% EDTA (Prevest Denpro, Jammu, India). All samples were irrigated with 5 ml of distilled water for one minute as a final rinse to remove any precipitate. The canals were dried with paper points (Densply-Mailiefer, Ballaigues, Switzerland) and the root apices were sealed with nail varnish. Each specimen was weighed using an electronic weighing machine (GR Series, A&D Company Ltd., Japan) for baseline weight.

Qualitative and quantitative analysis

The samples were numbered, and an intracanal medicament of silicone oil-based calcium hydroxide with iodoform (Metapex, Meta Biomed Co., Ltd., Chungcheongbuk-do, Korea) was placed in the prepared samples until the apex. These specimens were mounted in polyvinyl siloxane (Dentsply India Pvt. Ltd., Delhi, India) to keep them in a fixed position. No temporary restoration was placed as it may interfere with the quantitative assessment. The teeth were incubated at 37 °C and 100% relative humidity for seven days and weighed again for quantitative analysis.

The samples were segregated into four experimental groups on the basis of the irrigants used for the removal of medicament.

**Figure 1:**
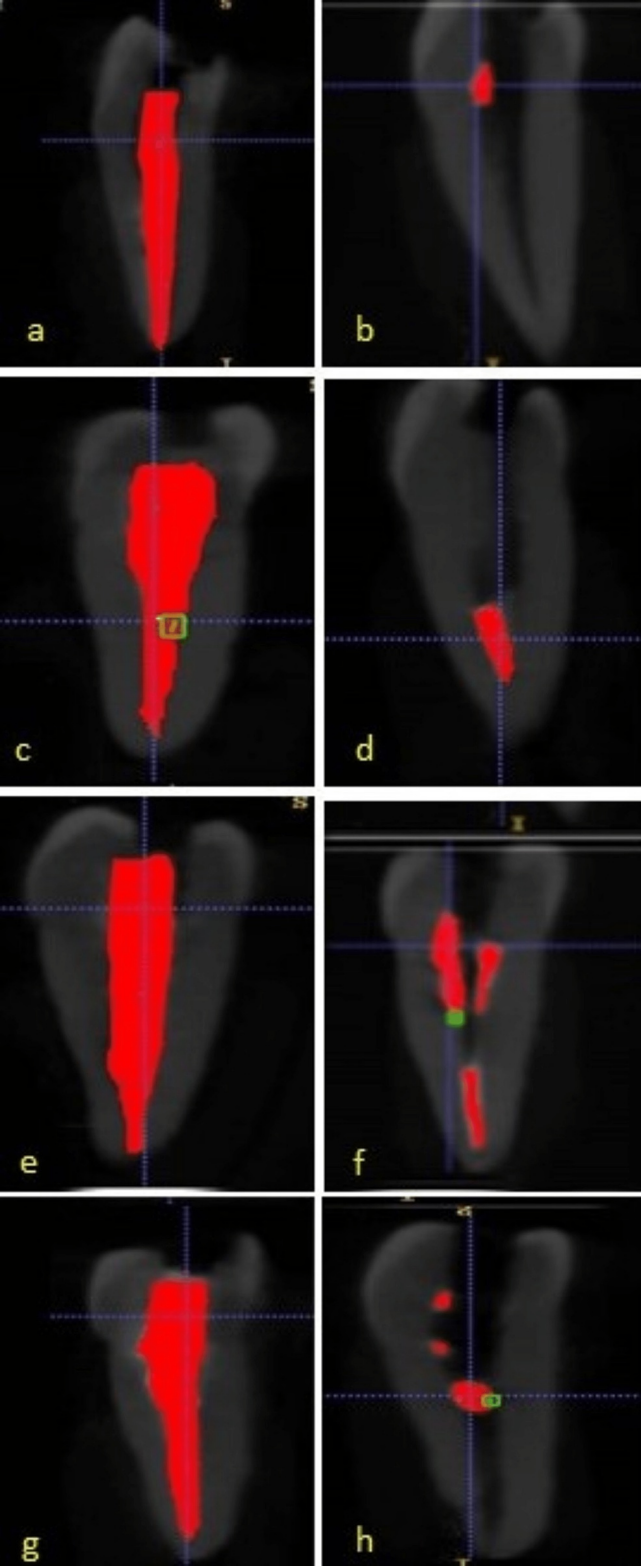
CBCT of teeth before and after removal of calcium hydroxide using the test agents. (a) CBCT of teeth filled with CH for 2% paradichlorobenzene (group 1); (b) CBCT of teeth showing remaining CH after treatment with 2% paradichlorobenzene + sonic agitation (group 1); (c) CBCT of teeth filled with CH for 10% citric acid (group 2); (d) CBCT of teeth showing remaining CH after treatment with 10% citric acid + sonic agitation (group 2); (e) CBCT of teeth filled with CH for 17% EDTA (group 3); (f) CBCT of teeth showing remaining CH after treatment with 17% EDTA + sonic agitation (group 3); (g) CBCT of teeth filled with CH for 0.2% Chitosan (group 4); (h) CBCT of teeth showing remaining CH after treatment with 0.2% Chitosan + sonic agitation (group 4).

Prior to the removal of medicament, cone beam computed tomography (CBCT) imaging (Carestream Kodak 8100, US) was done on all the samples for volumetric analysis. The data were exported as DICOM datasets. These data were then imported into the 3D image automatic segmenting and voxel-counting software ITK-Snap software (version 3.8.0 PICSL, U Penn, Pennsylvania, USA) for the calculation of the volume of the filled medicament in each sample (Figure 1a, 1c, 1e, 1g). The evaluations were conducted by an endodontist with knowledge of computed tomography and training in the use of the ITK-SNAP tools. The volumes of the segmented structures were measured in cubic millimetres (mm³).

The test irrigants for the removal of CH included 5 ml each of group 1: 5 ml of 2% paradichlorobenzene (ceruminolytic agent) (Clearwax, Cipla Ltd., Mumbai, India); group 2: 5 ml of freshly prepared 10% citric acid [10 g of citric acid monohydrate powder (Labogens, Ahmedabad, India)] with 100 ml of distilled water; group 3: 5 ml of 17% EDTA; and group 4: 5 ml of 0.2% Chitosan solution [prepared with 0.2 g of Chitosan (Acros Organics US, Powai, India; 90% degree of deacetylation)] diluted with 100 ml of 01% acetic acid. The mixture was stirred for two hours using a magnetic stirrer. The pH of the solution was adjusted to 3.2 [9].

A 30-gauge side-vented needle (DispoVan, Hindustan Syringes and Medical Device Ltd., Bihar, India) with the irrigant was placed 2 mm from the working length for delivering the agent to the canal. The test irrigants were agitated with EndoActivator (Dentsply Tulsa Dental Specialties, Tulsa, OK) for one minute and finally rinsed with 5 ml of distilled water.

**Figure 2:**
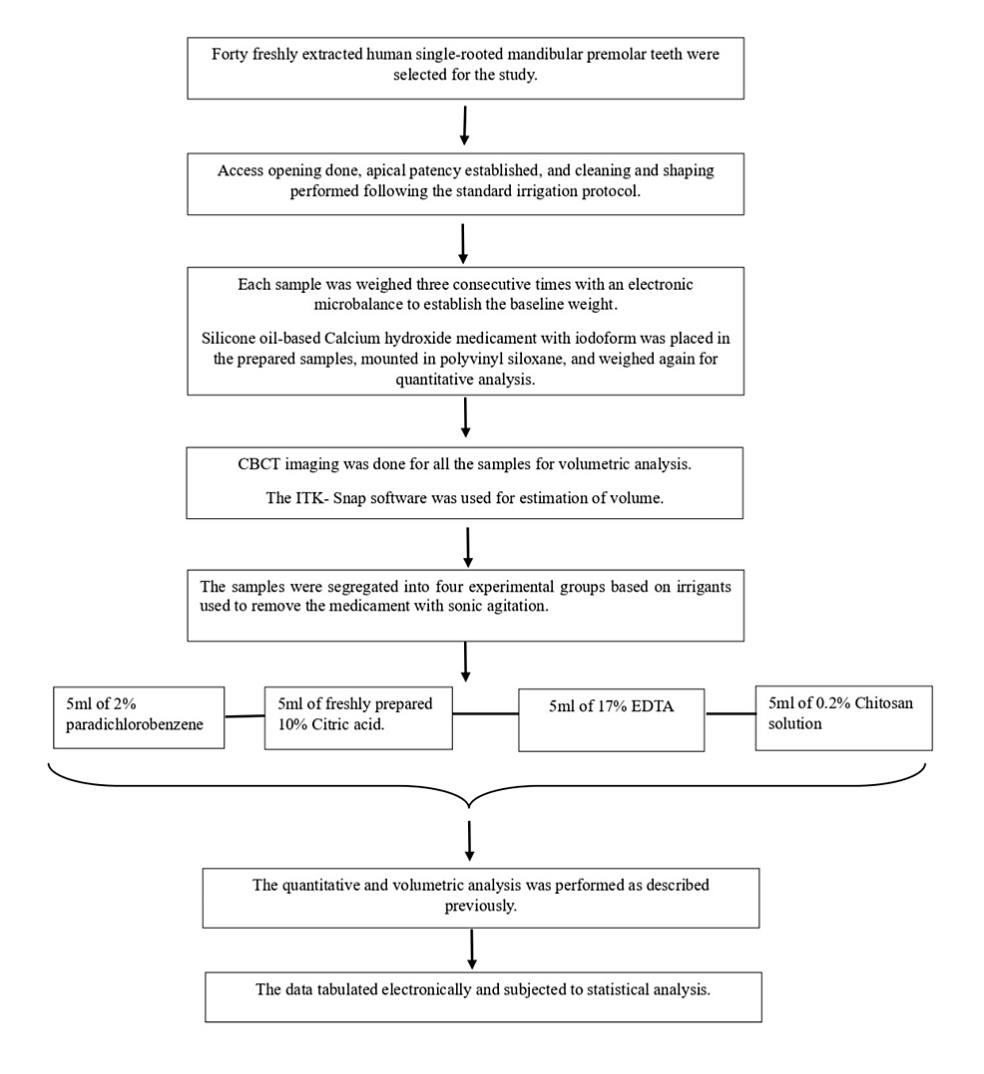
Flowchart for methodology.

After the removal of CH, all the samples were weighed again, and a second CBCT was performed to estimate the volume of the residual CH using ITK-Snap software (version 3.8.0 PICSL) (Figure 1b, 1d, 1f, 1h). All the data was recorded on MS Excel (Microsoft® Corp., Redmond, WA) and subjected to statistical analysis. The study design and flowchart for methodology are depicted in Figure 2.

Statistical analysis

The data were analyzed using SPSS version 26 (IBM SPSS, Inc., Chicago, IL, USA). The p-value of significance was set at 0.05. The confidence level was set at 95%, and the power of the study was fixed at 80%.

An analysis of variance was used to determine if the four groups had any difference in the volume and weight measurements after CH was removed. A Tukey post-hoc test was performed to compare each group to the other pair-wise.

## Results

**Figure 3:**
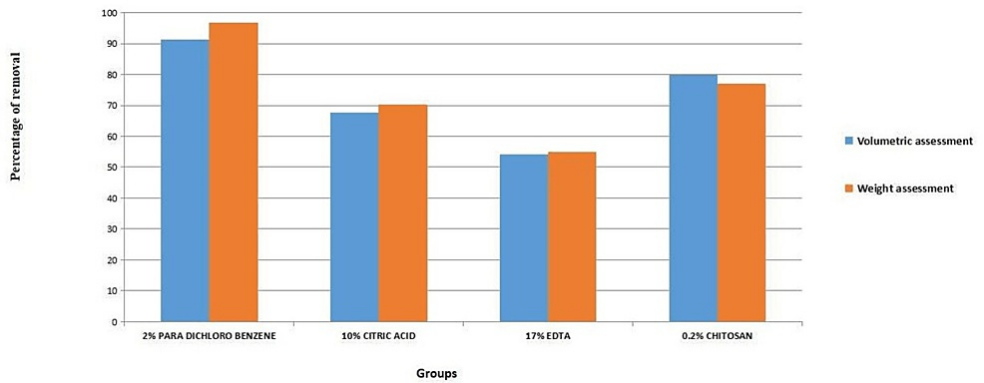
Percentage of calcium hydroxide removed using test irrigants by volumetric and weight assessment.

The mean percentage of CH removed with the various test irrigants is tabulated in Tables 1-2, respectively. There was a highly significant difference amongst the four groups in the mean percentage of CH removal through volumetric and weight assessment. The highest removal of CH was observed with 2% paradichlorobenzene, followed by 0.2% chitosan, 10% citric acid, and 17% EDTA, respectively, as shown in Tables 1-2 and Figure 3.

**Table 1:** Analysis of variance showing inter-group comparison of the four groups with respect to the difference of the filled and removed material in mg, percentage of removal and the minimum, maximum, mean and standard deviations with the relevant p-value. EDTA: ethylenediaminetetraacetic acid; p: level of significance. *p<0.05 is statistically significant, **p<0.01 is highly significant; an ANOVA test applied.

Variable	Number		Mean	Percentage of removal (%)	Std. deviation	Minimum	Maximum	F-value	p-value
2% paradichlorobenzene		10	39.12	96.75	14.47	19.38	68.95	4.55	0.00**
10% citric acid		10	25.88	70.24	7.35	12.98	36.29		
17% EDTA		10	23.57	54.95	7.49	12.17	33.82		
0.2% chitosan		10	27.97	76.91	9.92	14.04	47.22		

**Table 2:** Analysis of variance showing inter-group comparison of the four groups with respect to the difference of the filled and remaining volume, percentage of removal and the minimum, maximum, mean and standard deviations with the relevant p-value. EDTA: ethylenediaminetetraacetic acid; p: level of significance. *p<0.05 is statistically significant, **p<0.01 is highly significant; an ANOVA test applied.

Variable	Number	Mean	Percentage of removal (%)	Std. deviation	Minimum	Maximum	F-value	p-value
2% paradichlorobenzene	10	5.32	91.42	1.35	3.50	7.68	4.18	0.01*
10% citric acid	10	3.57	67.64	1.02	1.31	4.77		
17% EDTA	10	3.62	54.21	1.31	1.48	5.56		
0.2% chitosan	10	4.44	79.8	1.39	2.22	6.36		

Further analysis for pairwise comparisons was done using the Tukey post-hoc test, which showed that there was a significant difference between 2% paradichlorobenzene and all three groups for both volume (p=0.01) and weight (p=0.00) analyses. A comparison of 0.2% chitosan and 10% citric acid showed no statistically significant difference. All agents showed significantly higher removal of CH in comparison to 17% EDTA (p<0.05).

## Discussion

Intracanal medicaments used in clinical practice should be easy to introduce, have good antimicrobial activity, ensure effective sealing, and be easily removed from the root canal system. Calcium hydroxide is the most commonly used medicament, and its efficiency is influenced by the carrier vehicle [12,16,17]. Amongst the vehicles, oil-based formulations are difficult to remove from the canals compared to aqueous types [9-11,17]. The residue of CH prevents the adhesion of sealers to the dentinal tubules and interferes with sealing.

The present study aimed at assessing the removal of CH in an oily vehicle (CH + iodoform + silicone oil) by 2% paradichlorobenzene, 10% citric acid, 17% EDTA, and 0.2% chitosan when combined with sonic activation. The results demonstrated that 2% paradichlorobenzene was significantly more effective in removing CH from the root canal system. All the tested groups performed better than the EDTA group. Hence, the null hypothesis was rejected.

The carrier vehicles are aqueous, oily, and viscous. Calcium hydroxide with iodoform and silicone oil is non-water-soluble, which promotes low solubility and high diffusion of the paste within the tissues [4].

Many authors have studied the removal of aqueous, viscous, and oil-based CH from the root canal. They found 0.2% chitosan, followed by 10% citric acid, 7% maleic acid, and 17% EDTA, effective in removing oil-based CH [9,12,13]. They reported that, though aqueous and viscous-based preparations were retrieved from the canal, all the agents were unsuccessful in removing oil-based CH. Silicone-oil-based CH from root canals was selected based on its popularity of usage and difficulty of removal.

Paradichlorobenzene (2%) is a cerumen (ear wax) softening agent, and it helps to soften, disperse and dissolve the earwax [15]. The current study evaluated its off-label use as an endodontic irrigant to remove CH from root canals. It compared it to frequently recommended agents like citric acid, EDTA, and chitosan.

The use of irrigants like sodium hypochlorite and EDTA in combination with agitation is a common method for the removal of intracanal calcium hydroxide [9]. However, this technique does not adequately remove CH.

Several agitation techniques are used to remove CH, like hand files, rotary files, canal brushes, irrigation syringes, Endovac, sonics, and passive ultrasonics [10,18,19]. Sonic irrigation has been used in this study because of its superiority in removing intracanal medicaments [18,19].

The remaining root canal filling material can be analyzed by sectioning and assessment under a scanning electron microscope (SEM) or stereomicroscope [10,11,20]. These techniques may result in the loss of material and influence the result [21]. Moreover, they only allow the analysis of the surface area covered, not the volume. These drawbacks can be overcome by the use of non-invasive, three-dimensional imaging techniques (CBCT, microcomputed tomography) [9,13] and weight assessment [21,22].

The present study used CBCT data to evaluate the remaining CH volume using ITK SNAP software. The ITK-SNAP software is free software validated by its developers as a tool used to segment structures in neuroimaging, forensic anthropology, the medical profession, and others [23,24]. This software enables three-dimensional segmentation and has been used in analysis with proven reliability and efficiency [23,24]. Several authors have used three-dimensional imaging methods to study the correlation between secondary dentin formation and age by analyzing the tooth volume/pulp volume ratio [25-27].

The quantitative evaluation of the remaining CH was performed by weighing samples before and after loading the CH, and again after removal by test irrigants [22].

The results of the present study demonstrate that 2% paradichlorobenzene was significantly most efficient in calcium hydroxide removal, followed by 0.2% chitosan, 10% citric acid, and 17% EDTA, by both volumetric (p=0.01) and weight (p=0.00) assessments. There were no statistically significant differences among the 0.2% chitosan and 10% citric acid groups, but all groups had a significant difference with 17% EDTA (P<0.05).

The CH could be disintegrated by 2% paradichlorobenzene, reducing its natural integrity and allowing the smaller disintegrated particles to be removed by the standard irrigating mechanism. Furthermore, it is not a chelating agent and will not adversely affect the tooth structure [15].

In the present study, 0.2% chitosan performed better than 10% citric acid and 17% EDTA solution in the removal of CH. This is in accordance with the results of previous studies [9,13]. Silva et al. studied 0.2% Chitosan for its smear layer removal efficacy, which shows a chelation activity similar to other endodontic chelating irrigants [28]. Though chitosan is prepared with 1% acetic acid, the study by Silva et al. confirmed that the chelation was due to its own properties and not to acetic acid [29].

Two theories explain the efficacy of chitosan: the bridge theory states that two or more amino groups of chitosan bind to the same metal ion, and the pendant theory states that one amino group is utilized in the binding and the metal ion is linked to the amino group like a pendant. Either of the two mechanisms could be responsible for the chelation of calcium ions in dentin, resulting in the depletion of inorganic matter from the smear layer [28].

In the present study, 10% citric acid performed better than 17% EDTA, which is similar to the findings of Nandini et al., who also reported that 10% citric acid was more efficient in the removal of CH. They attributed this to its superior chelating property [17]. Ballal et al. also concluded that 7% MA and 10% citric acid removed the CH mixture significantly better than 17% EDTA [12]. Citric acid can penetrate the silicone oil better and shows superior chelation to the calcium ions [9,12,17].

The chelation of calcium ions with 17% EDTA helps in the removal of the smear layer. Similarly, it chelates calcium from calcium hydroxide medicament. Studies have shown EDTA to be more erosive on the dentin than other irrigating agents [29,30]. Silicone oil, which was the oily vehicle in Metapex, might have restricted its dissolution and removal from the root canal by the tested chemicals.

The limitations of the present study were the selection of large single-rooted mandibular premolars; the results may vary in posterior teeth with narrow and curved canals due to the challenge of accessing the irrigating solution in the apical third of the canals. The study entirely depended on the radio-opacity found in the CBCT, and the remnants were not verified with a SEM. Weight assessment has the limitation that the change in weight may be influenced by some loss of tooth substrate from the samples [21]. Further research could focus on the biocompatibility, toxicity, and tissue-dissolving ability of paradichlorobenzene, along with its interactions with other irrigants.

## Conclusions

The removal of intracanal medicaments like calcium hydroxide is essential prior to obturation, as the remnants interfere with the setting of root canal sealers and cause microleakage. In the present study, 2% paradichlorobenzene, commonly used as a cerumenolytic, was tested for its off-label use for the removal of calcium hydroxide in comparison to 0.2% chitosan, 10% citric acid, and 17% EDTA.

Within the limitations of this study, 2% paradichlorobenzene in combination with sonic agitation was most efficient in the removal of calcium hydroxide, followed by 0.2% chitosan, 10% citric acid, and 17% EDTA.
